# Blame the Player, Not the Game? How Perceived Institutional Inequality Predicts Displaced Aggression

**DOI:** 10.3390/bs15121662

**Published:** 2025-12-02

**Authors:** Yang Fan, Shanghua Gong

**Affiliations:** School of Marxism, Hangzhou Normal University, Hangzhou 311121, China; 2023111016011@stu.hznu.edu.cn

**Keywords:** pro-authoritarian attitude, displaced aggression, institutional inequality, narrative interpretation

## Abstract

Why do individuals, when facing institutional injustice, direct their anger toward peers rather than powerful actors? The existing literature typically explains displaced aggression through emotional arousal or power asymmetries. However, we argue that interpretive meaning within specific institutional contexts plays a more decisive role in shaping aggressive behavior. Drawing on a triadic framework of structural stimulus, narrative interpretation, and behavioral response, we conducted a scenario-based survey of 1109 Chinese university students across five institutions. The results show that perceived institutional inequality significantly increases displaced aggression (β = 0.388, *p* < 0.001), but not upward aggression (β = 0.091, *p* = 0.061). Two mediating mechanisms, perceived cost of aggression and inequality justification, account for 15.3% and 12.4% of the total effect, respectively. Moreover, pro-authoritarian attitude significantly amplifies the effect of perceived inequality on displaced aggression (interaction β = 0.224, *p* < 0.001). In addition, we find a counterfactual result that females show 0.248 units more displaced aggression than males under perceived inequality. These findings highlight how individuals internalize inequality as meaningful and actionable, even in constrained political settings. This study contributes a narrative-based theoretical framework for understanding misdirected aggression under institutional inequality.

## 1. Introduction

From the surge in domestic violence during COVID-19 lockdowns to the rising prevalence of school bullying and online harassment, displaced aggression has become a global social issue that transcends culture and geography. While many individuals express frustration directly at their sources of dissatisfaction, others redirect their hostility toward uninvolved or vulnerable targets. This behavioral puzzle raises a critical question: When individuals experience structural injustice, why do they sometimes choose to attack weaker peers instead of confronting those in power?

Previous research has traditionally explained displaced aggression through the frustration–aggression hypothesis (Dollard et al., 2013), similarity theory (Foster et al., 2005), and emotional dysregulation frameworks (Berkowitz & Harmon-Jones, 2004; A. Zhang & Zhang, 2023). Scholars have also distinguished it from upward aggression by emphasizing factors such as target submissiveness (Lagios et al., 2025), low reciprocity, and power distance (Ettekal & Ladd, 2020). However, current explanations either focus on proximal stimuli or structural constraints, with insufficient attention to how individuals interpret and justify aggression within institutional contexts. Our understanding of when and why displaced aggression arises remains incomplete without considering the role of narrative-based cognition and the perceived cost–benefit calculus of aggression. This gap risks conflating symbolic compliance with passive submission, and misjudging aggression as a purely emotional outburst rather than a cognitively rationalized act.

To bridge this gap, our study introduces a triadic framework that connects institutional structures, individual cognition, and behavioral outcomes. Guided by the Narrative Policy Framework (NPF) (McBeth et al., 2014) and cognitive theories of interpretive intention (Bruner, 1991; Kahneman, 2013), we treat perceived institutional inequality as a structural trigger. Its effects on behavior depend on how individuals make sense of it. Specifically, we examine how perceived legitimacy and perceived costs influence responses to institutional inequality, and how ideological tendencies shape and moderate these reactions, such as pro-authoritarian attitude (Jost et al., 2009).

To explore these questions, we conducted a scenario-based survey with 1109 university students from five institutions across eastern, central, and western China. The sample included both elite and non-elite universities, ensuring institutional diversity and regional variation. Our measurement strategy combined general attitude items with context-rich behavioral scenarios to strengthen both internal and external validity.

This study aims to contribute to the existing literature in several ways. First, it conceptualizes displaced aggression as a meaning-driven behavioral response to perceived institutional inequality, rather than a purely reactive impulse. Second, it links macro-level institutional factors with micro-level cognitive processes to explore the mechanisms behind displaced aggression, particularly the perceptions of legitimacy and cost. Third, it explores how individual political dispositions moderate these effects, especially pro-authoritarian attitude. Fourth, the study offers a gender-sensitive perspective to understand the differential expression of displaced aggression.

The remainder of this paper is organized as follows. Section 2 reviews theoretical foundations and presents four hypotheses. Section 3 outlines the data, measures, and analytical strategy. Section 4 reports empirical findings. Section 5 discusses theoretical implications, gender and ideological dynamics, and study limitations. Section 6 concludes with key insights on displaced aggression as a narrative response to perceived institutional inequality.

## 2. Literature Review

### 2.1. From Aggression to Displaced Aggression

The concept of displaced aggression originates from the broader construct of aggressive behavior. In social psychology, aggression is defined as behavior directed at an individual or group with the intent to cause harm, whether physical, verbal, relational, or symbolic (Anderson & Bushman, 2002; Baron & Richardson, 1994). Unlike proactive aggression, which serves as a tool (Buss, 1962) or unintentional harm, displaced aggression is viewed as a reactive action, primarily driven by anger, retaliatory motivation, and frustration (Dollard et al., 2013; Lagios et al., 2025). It is assumed that aggression is a response to a previous offense, rather than being initiated by an active aggressor. Hence, drawing on Lagios et al. (2025), we conceptualize displaced aggression as retaliatory behavior directed at an uninvolved individual, rather than the original source of provocation.

In addition to the biological perspectives (Archer, 2004; Crick & Grotpeter, 1995; Tedeschi & Felson, 1994), classic theoretical accounts rely on similarity theory to explain the origins of displaced aggression. According to this view, if the new target shares similarities with the source of frustration, such as appearance or proximity, these cues may trigger an aggressive response. However, in recent years, scholars have argued that perceived similarity cues do not necessarily lead to corresponding behaviors, especially since aggressive behavior carries a high risk of social norm violations (Foster et al., 2005). Therefore, similarity serves as a background condition, while the actual drivers of displaced aggression are individuals’ behavioral tendencies and personality traits within that environment. As a result, scholars have further supplemented their understanding by examining emotional states, cognitive abilities, and behavioral imitation (Berkowitz & Harmon-Jones, 2004; Denson et al., 2009; Kaluza et al., 2020; A. Zhang & Zhang, 2023). For example, A. Zhang and Zhang (2023) found that adolescents are more likely to misattribute the source of their frustration when experiencing intense emotional dysregulation.

Previous studies have often focused on differences in the forms of displaced aggression. For instance, triggered displaced aggression differs from pure displaced aggression in that it involves strong reactions to minor provocations (Navas-Casado et al., 2023). This is often attributed to a synergistic effect between the minor cue and the lingering frustration from the original incident (Panter-Brick, 2023). Such research provides general insights, particularly in clarifying the conceptual boundaries and underlying mechanisms of displaced aggression. However, an important factor has been overlooked: the target. Which individuals are chosen as targets of displaced aggression, and which are not, remains an underexplored question.

### 2.2. Displaced Aggression vs. Upward Aggression

The existing literature generally holds that secondary targets often possess traits that make them more susceptible to attack, such as high submissiveness, that increase their vulnerability to aggression (Lagios et al., 2025; Mahadevia et al., 2021). Building on this idea, some scholars argue that weak reciprocal relationships with groups contribute to displaced aggression, such as minorities. They also suggest that poor regulation of negative stimuli plays a key role, as seen in adolescent bullying (Ettekal & Ladd, 2020). Overall, high submissiveness conveys a perceived image of passivity, inferiority and low likelihood of resistance, and this image may not always reflect reality (Greitemeyer & Sagioglou, 2016). In such context, this helps explain why upward aggression is often suppressed, whereas displaced aggression emerges as an alternative. For example, in hierarchical systems and organizations, leaders are often idealized and positioned beyond reproach (Tan, 2020). The pressure they exert on subordinates or the general public is often redirected toward peers or more vulnerable individuals, rather than back toward the leaders themselves.

However, theories based on relative power comparisons do not support a stable shift from upward to displaced aggression. First, empirical evidence shows that individuals with limited power are not always passive; marginalized groups may resist their direct superiors, as demonstrated in various protest movements (Dai & Spires, 2018). Furthermore, this body of literature often conflates high power positions with institutional unresponsiveness (Zheng & Meng, 2021). The lack of upward aggression may stem not from fear of authority or potential punishment, but from the absence of effective channels or a belief that resistance would be futile. As a result, aggression is redirected toward individuals or groups who appear submissive but are closely connected to the aggressor (Reijntjes et al., 2013). This pattern is especially common in organizational settings. Thus, relative power differentials may not be a necessary condition for displaced aggression to occur.

While power comparisons do not reliably lead to displaced aggression, we argue that the emergence of aggressive behavior hinges on how individuals subjectively interpret the meaning of their actions. Cognitive science suggests that, whether in the case of displaced or upward aggression, acting on aggression requires more than merely perceiving a target as ‘attackable’ (Surdu et al., 2021). It also requires the internalization and interpretation of the act’s meaning. Without this process, perceptual cues alone are insufficient to trigger aggressive behavior. This perspective helps illuminate the psychological mechanism behind the ‘banality of evil’, explaining how individuals like Eichmann could participate enthusiastically in the mass murder of Jews without perceiving their actions as morally wrong (Minnich, 2014).

In short, whether rooted in structural power disparities or micro-level perceptions of submissiveness, aggression only emerges when individuals adopt a narrative, assign meaning to their actions, and are subsequently motivated and conditioned to act (Elcheroth & Reicher, 2017). It is the interpretation of the situation not the stimulus itself, that fundamentally drives the occurrence of displaced aggression. However, current research on the situational interpretation of aggressive behavior remains limited. Nevertheless, the Narrative Policy Framework (NPF) theory offers a valuable analytical framework for understanding such processes (McBeth et al., 2014). Based on the assumption of the concept of homo narrans (Rabatel, 2008), the idea that humans are inherently storytelling beings, it suggests that public narratives can portray a specific group as exploiters while casting the government (or the institutional system) as a herotic protector (Shanahan et al., 2013). This framing discourages upward resistance and redirects aggression toward particular groups as targets of displaced aggression. Building on this theoretical framework, the next section will explore potential debates and present the hypotheses of the current study.

### 2.3. Factors and Mechanisms Influencing Forms of Aggression

The narrative hypothesis suggests that, although causality is abstract, internalized narrative structures help individuals organize fragmented information into a coherent causal logic that guides their behavior (Bruner, 1991). To explain why displaced aggression occurs instead of upward aggression, it is necessary to build an integrated framework for analyzing possible narrative structures. Given that both a primary target (such as those in power), and a secondary target (such as peers or subordinates) are involved, a triadic structure is needed. This structure consists of the state (system), the individual, and the other (society).

Traditional narrative perception theory outlines static roles such as the hero, victim, and villain (Shanahan et al., 2013). However, we argue that displaced aggression tends to emerge in contexts marked by institutional deprivation and perceived injustice. In such contexts, individuals begin to see themselves as victims within a broader narrative. They assign the roles of hero and villain to their superiors and to their peers or subordinates, thereby displacing rather than directing their aggression upward.

Research has shown that institutionalized inequality is most likely to generate structural resentment and aggressive impulses. This form of inequality refers to individuals’ subjective perceptions regarding the unjust distribution of resources, institutional procedures, and opportunities accessibility of opportunities provided by the system (Colquitt, 2001; Lind & Tyler, 1988). It captures a macro-level psychological construct that integrates personal experiences with broader social structures. Unlike concrete instances of interpersonal fairness, perceived institutional inequality serves as a cognitive schema that shapes individuals’ interpretations of institutional legitimacy and their own place in the social hierarchy. According to relative deprivation theory, individuals who perceive inequality through peer social comparison are prone to feel resentment, frustration, and hostility. These emotions may in turn increase the likelihood of aggressive behavioral responses (Smith et al., 1993). However, individuals rarely confront structural sources of inequality directly, especially when those sources are powerful, abstract, and unchangeable. As a result, aggressive tendencies may be redirected toward more accessible or weaker targets, forming a pattern of displaced aggression (Hoobler & Brass, 2006; Liu et al., 2015). Therefore, we hypothesize that:

**H1.**  *Perceived institutional inequality positively predicts displaced aggression.*

Building on the theory of situational interpretation, we propose that perceived institutional inequality contributes to increase displaced aggression and reduce upward aggression by facilitating interpretive justifications that legitimize aggressive responses. Previous research on self-justification has largely emphasized the role of negative emotional arousal. For example, Greitemeyer and Sagioglou (2016) demonstrated that institutional inequality increases aggressive behavior by eliciting anger and shaping behavioral intentions. Similarly, J.-B. Li and Finkenauer (2023) found that institutional distrust fosters displaced aggression through heightened hostility toward outgroups.

However, we argue that because aggression inherently violates social norms and entails high social costs, emotional impulses are insufficient to produce behavioral outcomes. These impulses may still involve cognitive processing, but they rarely translate into action. For instance, disliking a political candidate does not necessarily translate into an attack against them.

Instead, we contend that the intent-based justification rooted in interest framing is the real driver of aggressive behavior. Research in management and political science suggests that framing based on perceived costs (benefits) of related behavior is more effective in motivating behavior than alternative framing strategies (Gavrilets, 2015; Shao & Liu, 2019).

Based on this reasoning, we argue that perceived institutional inequality leads individuals to believe that targeting those in lower social status involves relatively low costs. Thus, we hypothesize that:

**H2.**  *Perceived institutional inequality is related to higher levels of displaced aggression via the awareness of lower aggression cost.*

Moreover, we also argue that perception of institutional inequality may suggest a cognitive interpretation that “inequality is universal and justifiable” which in turn legitimizes aggression against those with lower social status. Although legitimacy is a highly abstract concept and less intuitive than cost–benefit perceptions, evidence shows a strong perception of legitimacy can be more effective in motivating individuals who are hesitant or indecisive (Berejikian, 1992). Therefore, we hypothesize another mediation mechanism that:

**H3.**  *Perceived institutional inequality is associated with displaced aggression via the inequality justification.*

As a form of hot cognition, interpretive intention reflects immediate and impulsive psychological responses. Scholars have found that such cognition plays a significant role in quick-response scenarios, reflecting the System 1 thinking (Kahneman, 2013). In contrast, human cognition also involves slower and more analytical processing, through which ideologies exert a rational and long-term influence on behavior (Jost et al., 2009). This is a comprehensive knowledge framework that deeply and rationally influences people’s behavior.

Building on this theoretical background, the present study examines these mechanisms within a Chinese sociocultural context, providing an opportunity to explore how the general pathway from perceived institutional inequality to displaced aggression functions under specific cultural assumptions. Unlike other societies and cultures, contemporary Chinese culture, rooted in Confucian collectivism, emphasizes moral self-restraint, relational harmony, and deference to authority. Such orientations socialize individuals to internalize frustration and seek moral or symbolic justification rather than direct confrontation (Fry, 2017). Consequently, aggression tends to be cognitively reframed and redirected toward safer or socially acceptable targets, an interpretive process that rationalizes displacement as a form of moral defense rather than deviant behavior. Therefore, we argue that ideological preferences that prioritize institutions, systems, or higher authorities, while devaluing those with lower social status, contribute to the emergence of displaced aggression. For example, pro-authoritarian attitude is one ideology that reinforces such tendency.

According to conventional view of social psychology, pro-authoritarian attitude refers to an orientation characterized by submission to authority (Adorno et al., 1993). Practically, those individuals with pro-authoritarian attitude tend to support repressive rule and favor elitism (Duckitt & Sibley, 2007; McFarland, 2005). Recently, scholars have found evidence that pro-authoritarian attitude may also lead to social dominance orientation, because they often internalize the values of authoritarian systems, typically xenophobic ones, and view out-group “others” as threats (Crawford et al., 2016; Van Hiel et al., 2020; Zubielevitch et al., 2023). Such views often focus on hostility between different ethnic groups. However, we argue that this internalization is not only racial, but also class-based. This is because institutional inequality implies a right-wing value rooted in elitism, which glorifies the resource dominant individuals or groups, but it does not necessarily entail racist assumptions. Based on this, we hypothesize that:

**H4.**  *Individuals with pro-authoritarian tendencies are more likely to engage in displaced aggression.*

**Figure 1:**
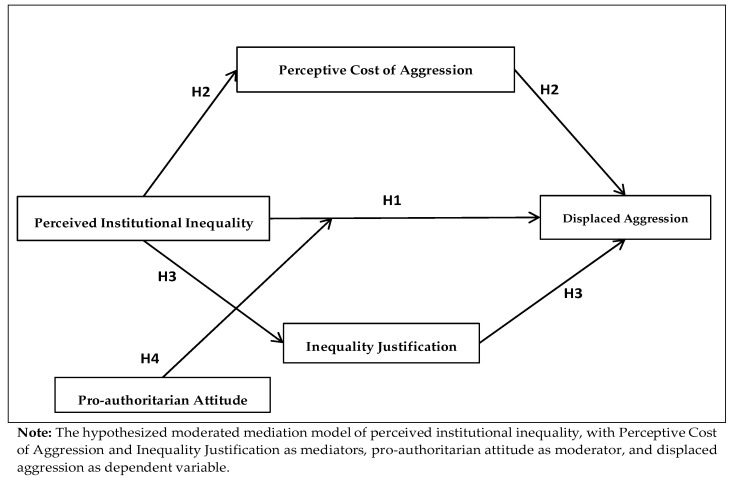
Hypothesized Moderated Mediation Model.

Our study aims to construct a more comprehensive theoretical framework for understanding the emergence of displaced aggression. It incorporates perceived institutional inequality as a structural explanatory variable, reflecting institutional deprivation. At the same time, the framework integrates cognitive mechanisms, including interpretive intent and ideological orientation, to supplement structural factors with micro-level insights. This multi-level approach enhances the theoretical framework’s explanatory power. Figure 1 shows our research hypothesis model.

## 3. Methods

### 3.1. Sample Selection and Justification

This study selected university students in mainland China as the target population, based on both cultural and theoretical relevance for three main reasons. First, Chinese society is typically characterized by Confucian collectivist values that emphasize affective restraint and hierarchical social order, thereby fostering indirect expressions of discontent to maintain relational harmony (Markus & Kitayama, 1991; Matsumoto et al., 2008). Compared to other collectivist societies such as Japan and South Korea, institutional structures in China tend to discourage formal avenues of protest or the expression of bottom-up dissatisfaction (Minzner, 2006), and instead promotes the resolution of grassroots grievances through local, informal mechanisms rather than through formal channels such as public criticism, protests, legal action, or direct confrontation (Jiang & Xie, 2020). Within China’s high power distance structure, the existence of authority is highly internalized among the citizens, rendering disengagement from public procedure and apathy from public expression (Shao & Liu, 2019; W. Zhang, 2022). Scholars have also observed that Chinese citizens are embedded in institutional contexts characterized by intensified top-down organizational pressures, which shape their everyday experiences and normative expectations (Farh et al., 2007; Zhu et al., 2024).

Second, the university environment closely mirrors institutional inequality, as it reproduces key structural inequalities in both institutional design and student composition. On the one hand, mechanisms such as scholarship distribution and graduate school admissions in China are not always fully transparent or based on open competition. On the other hand, students come from diverse family backgrounds, reflecting disparities in social class, resource accessibility, and social status (Banales et al., 2022). We argue that such environmental characteristics enable student populations to develop a more nuanced understanding of the concept of inequality, thereby reducing potential biases.

Third, the university setting in China, relatively speaking, provides an environment of free choice, which helps avoid the potential interference of fixed patterns shaped by a single context. If compared to other public spheres such as external public network, it has relatively fewer institutional constraints on bottom-up grievance (Yu, 2022). The internal university forums and communication channels, though semi-enclosed, allow comparatively greater space for expression and deliberation. This relative openness explains why many upward challenges to authority in China, such as the #MeToo movement against faculty sexual misconduct, have emerged from university campuses (Ling & Liao, 2020). The campus environment thus provides individuals with a higher degree of perceived autonomy, making behavioral patterns more variable and responsive, rather than fixed.

Building on the three points above, we argue that Chinese university students constitute a theoretically appropriate and empirically valuable population for investigating our research questions.

The online survey was administered through a structured questionnaire hosted on a secure survey platform. The invitation link was distributed via university forums in several universities across different regions of China. Participation was voluntary, and a small completion reward was provided to encourage engagement. In line with standard ethical guidelines for online research, the introductory page clearly described the study’s purpose, procedures, and voluntary nature, emphasizing that responses would be used solely for academic research. Participants were assured of full anonymity, as no identifying information, such as names, student identification numbers, and IP addresses was collected. After reading the introduction, respondents were required to indicate informed consent by clicking the button labeled “I agree to participate in this survey,” before accessing the questionnaire items.

**Figure A1:**
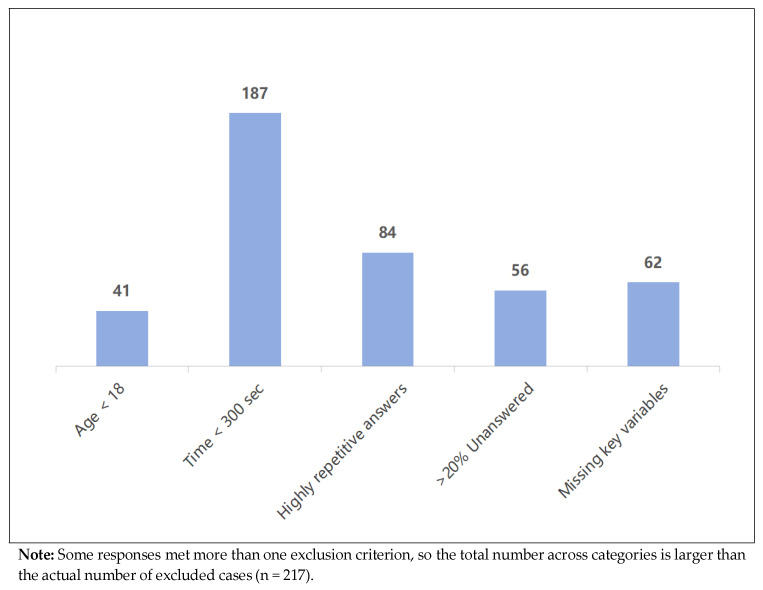
Number of Cases Excluded by Data Cleaning Criteria.

By means of an online investigation, we surveyed 1326 university students from five institutions across eastern, central, and western China, encompassing both elite and non-elite universities. The sample included both undergraduate and graduate students. All participants completed an anonymous online questionnaire after providing informed consent. The study complied with standard ethical guidelines, ensuring anonymity and confidentiality. Responses were excluded based on the following criteria: (1) the respondent was under 18 years old; (2) the completion time was less than 300 s; (3) more than 20% of responses exhibited strong repetitive patterns; (4) more than 20% of the questions were left unanswered; or (5) key variables for dependent or independent measures were missing. After data filtering and cleaning, we retained 1109 valid observations for analysis. No significant differences were found between the retained and excluded cases in terms of gender, age, or study level, indicating that the final sample is demographically consistent with the original dataset. The distribution of excluded responses across the five criteria is illustrated in Appendix A Figure A1, where overlapping exclusions explain why the total count exceeds the number of unique excluded cases.

In the final sample, 37% of participants were female. Although this proportion appears lower than national averages, it reflects the composition of the sampled institutions and disciplines, particularly those with male-dominated majors such as engineering and politics. Gender was controlled in all subsequent analyses to account for its potential influence.

### 3.2. Measurements

We designed the questionnaire by introducing scenario-based items. Compared to traditional questionnaires which are designed to measure general attitudes through decontextualized items (Christensen et al., 2020), scenario-based questions are more effective in capturing respondents’ behavioral preferences within given context (Rocha Flores et al., 2014). In our study, we aim to examine not only the effect of general attitudes to ensure external validity, but also the influence of contextualized stimuli to enhance the internal validity. Therefore, for both the dependent and independent variables, we employed two types of questionnaire items: general attitude items and scenario-based items to measure each construct. After confirming that both sets met the minimum reliability threshold (Cronbach’s α ≥ 0.70), we computed the mean score of all items within each set to create composite variables representing the corresponding constructs. Respondents were encouraged to reflect on the frequency of relevant behaviors when answering the items, which were measured by 5-point Likert scales ranging from “very unlikely” to “very likely,” for dependent or independent variables; or “strongly disagree” to “strongly agree” for mediator or moderator variables, as detailed below. A pilot test was conducted before the formal survey (150 distributed, 127 valid responses), and the results showed acceptable reliability and validity (Cronbach’s α = 0.77–0.89; KMO = 0.82–0.93). These indices were calculated separately for each scale and are consistent with those reported in prior Chinese validation studies.

**Displaced Aggression (dependent variable).** Drawing on established scales of displaced aggression and grounded in frustration-aggression theory (Berkowitz, 1989; Denson et al., 2006; Martinko et al., 2002; Su & Xia, 2020), we adopted their scales, including six items totally (such as “*You express anger toward the people with lower social status even when they are not the cause of your anger*.”). The Cronbach’s α for this scale is 0.73, showing good internal consistency.

To reduce social desirability and acquiescence bias and enhance the robustness of our findings, we employed **a pairwise questioning strategy** (Kam & Meyer, 2015; Kuru & Pasek, 2016). In addition to measuring displaced aggression, we included the **upward aggression** as comparative alternative for displaced aggression. In this study, protest was used as an operational indicator of upward aggression because it represents a common and socially recognized expression of confronting authority (L. Li, 2021), and it allows for greater privacy and lower non-response rates compared to more extreme forms like hunger strikes. We adopted their scales, including four items totally (such as “*If you suffer actual losses due to an unequal institutional rule, you will protest the government in public (either verbally or through actions)*.”). The Cronbach’s α for this scale is 0.85, showing high internal consistency.

**Perceived institutional inequality (independent variable).** We measured this construct primarily using items from the Chinese General Social Survey (CGSS 2021) with additional reference to established measures in previous research (Colquitt, 2001; Tyler & Blader, 2013). Specifically, instead of asking respondents whether they felt institutional equality as traditional scholars did, we directly asked five items about experiences of unequal institutional arrangements in a more intuitive and respondent-friendly manner (such as “*The government prevents the fair distribution of resources.*”). The scale showed good internal consistency (Cronbach’s α = 0.76).

**Interpretation of Aggression (mediator variables).** We have two mediator variables: inequality justification and perceptive cost of aggression. Based on the aggression questionnaire (Buss & Perry, 1992; Maxwell, 2007), we adapted and designed three scenario-based items to assess the meaning of behavior. Respondents rated the extent to which they agreed with the following statements of the **Awareness of Aggression Cost** (such as “*If a higher social status person (*e.g.,* the rich) attacks a lower social status person (*e.g.,* the poor), the higher-status person is able to use their means to escape punishment.*”). The scale showed high internal consistency (Cronbach’s α = 0.81). An exploratory factor analysis showed a clear single factor structure (KMO = 0.875, Bartlett’s χ^2^ (45) = 1547.84, *p* < 0.001, All loadings > 0.70).

For **inequality justification**, we also adopted three items from egalitarianism scale (Jingjing et al., 2023; Martin & North, 2022), for example, “*You believe that some people should enjoy privileges while others should not.*” The scale showed good internal consistency (Cronbach’s α = 0.76). The scale showed good internal consistency and a clear one factor structure (Cronbach’s α = 0.76, KMO = 0.89, Bartlett’s χ^2^ (21) = 4105.83, *p* < 0.001, All loadings > 0.73).

**Pro-Authoritarian Attitude (moderator variable).** This construct was measured using items adapted from established authoritarian personality scales (Ma & Wang, 2015; Nilsson, 2024). We adopted their scales, including five items totally (such as “Everyone should unconditionally obey their superiors”). The Cronbach’s α for this scale is 0.77, showing good internal consistency.

**Control Variables.** Five demographic characteristics were included as control variables in the analysis. Three of them were binary variables: (1) Gender, coded as 1 for female. (2) Chinese Communist Party (CCP) membership (coded as 1 = party member). (3) Household registration (coded as 1 = urban resident). The other two are continuous variables. (4) Annual household income (in CNY) and (5) Age (in years). These variables were selected based on their relevance to group differences in the perception of institutional inequality and aggressive behavior, either due to biological factors or sociodemographic characteristics (Archer, 2004; Eagly & Wood, 2012). By statistically controlling for these variables, we aimed to reduce potential confounding effects and ensure more robust estimation of the hypothesized relationships.

To maximize the potential for inferring causal relationships from the survey data, we arranged the scale order based on the principle of temporal precedence in questionnaire design (Bechlivanidis & Lagnado, 2016; Fiedler et al., 2011), presenting items in the sequence of **independent variables, mediators, and dependent variables.** For the two parallel mediating variables, we employed matrix-style items to enhance clarity and consistency. To minimize potential order effects or priming induced by item placement, the items within each scale block were randomized.

### 3.3. Analysis Model

We employed OLS as the primary estimation method. Additionally, we used the Sobel-Goodman test to detect and analyze mediation effects. All control variables were included in each regression model. Robust standard errors were used to correct for heteroskedasticity and to improve the precision of the estimated coefficients (Stock & Watson, 2008).

## 4. Results

### 4.1. Preliminary Analyses

#### 4.1.1. Common Method Bias Test

To assess the potential influence of common method bias, Harman’s single-factor test was conducted following the procedure proposed by Podsakoff et al. (2003). All measured items were entered into an exploratory factor analysis with the extraction constrained to one factor. The results showed poor model fit for the single-factor solution (χ^2^ (595) = 14,000.00, *p* < 0.001; RMSEA = 0.110; CFI = 0.605; TLI = 0.575), indicating that no single common factor accounted for most of the variance. This suggests that common method bias is unlikely to pose a serious threat to the validity of the findings.

#### 4.1.2. Descriptive Statistics

The following Table 1a,b shows the descriptive statistics of variables of our research. Overall, participants report higher levels of displaced aggression (M = 4.21, SD = 0.99) while lower upward aggression (M = 2.43, SD = 0.80). The perceived institutional inequality (M = 3.32, SD = 0.57). For the mediator variables, both awareness of lower aggression cost (M = 3.05, SD = 0.96) and inequality justification (M = 2.50, SD =0.87) cluster around the neutral option. The average pro-authoritarian attitude shows slight disagree of this ideology among our participants (M = 2.74, SD = 0.97). The demographic results show among our sample, 37% are female, 10% are members of the Chinese Communist Party (CCP), the average age is 22, and the average annual family income is approximately 132,789 yuan. Additionally, 73% of participants come from urban backgrounds. These demographic characteristics are broadly consistent with the contemporary campus population in China (Huang, 2021; Xie & Jin, 2022).

### 4.2. Regression Results

#### 4.2.1. Main Effects (H1)

The following Table 2 shows that higher level of perceived institutional inequality significantly predicts more displaced aggression (β = 0.388, SE = 0.048, *p* < 0.001) in Column 1, while not encourages predict upward aggression. As shown in Column 2, the effect is not statistically significant (β = −0.091, SE = 0.049, *p* > 0.05).

This result indicates that higher levels of perceived institutional inequality are associated with an increased aggression against individuals perceived as lower in social status. The effect is particularly strong, because one-unit increase in perceived inequality corresponds to an increase of nearly 10% of a standard deviation in displaced aggression, relative to its mean (4.21). However, perceived institutional inequality does not significantly lead to upward aggression. This suggests that regardless of how unequal participants perceive the institution to be, they remain reluctant to publicly protest the superior. Therefore, the results support H1.

#### 4.2.2. Mediation Analysis (H2 and H3)

As shown in Table 3, the mediation effect of awareness of lower cost is 0.156, which is significant, which mediates 15.3% of the total effect. Interestingly, we found the cost-related mediator also contributes to the upward aggression, while mediates 23.7%. This implies that if institutional inequality is perceived as a signal of reduced aggression costs, it may not only reinforce downward aggression, but also generate spillover effects that lead to upward aggression. Although this finding contradicts our H2, we argue that it reveals an important mechanism. Specifically, the perception of lower aggression costs makes aggression against weaker targets seem feasible. Moreover, it implies that aggression against more powerful targets is also within reach.

For H3, we employed the same method. As presented in Table 4, the mediation effect of inequality justification is also significant, which is 0.155. This accounts for 12.4% of total effect. However, this mediating effect is only significant in the case of displaced aggression, and does not hold for upward aggression. We argued that the perception of the legitimacy of aggressive behavior serves as the key mechanism underlying displaced aggression specifically targeted at individuals of lower social status. Hence, these results strongly support H3.

#### 4.2.3. Moderation Analysis (H4)

For H4, the interaction term between perceived institutional inequality and pro-authoritarian attitude was included in the regression models (see Table 5). We found a significant effect of the interaction term in the Column 1 (β = 0.224, *p* < 0.001), while Columns 2 shows no significant effects when the dependent variable is upward aggression. In other words, individuals with stronger pro-authoritarian attitude exhibit significantly higher levels of displaced aggression under conditions of perceived institutional inequality. These results support H4.

### 4.3. Additional Finding (Gender’s Moderating Effect)

We further found that gender (female = 1) moderates the relationship between perceived institutional inequality. As shown in Table 6, under conditions of perceived institutional inequality, female participants exhibit 0.248 unit more displaced aggression (Column 1) than male participants. However, there is no significant gender difference in the likelihood of engaging in upward aggression (Column 2).

Additionally, several **robustness checks** were conducted to ensure the reliability of the findings. Alternative model specifications and variable substitutions do not substantially alter the core results, confirming the stability and robustness of the main conclusions. Detailed results are presented in the Appendix C.

## 5. Discussion

In this study, we examine how perceived institutional inequality influences individuals’ aggressive responses in the university setting of China. Our findings suggest that perceived institutional inequality is positively associated with displaced aggression toward peers, rather than with upward aggression. Furthermore, this relationship is strongly mediated by individuals’ awareness of lower aggression cost, and amplified by a pro-authoritarian attitude, which strengthens the tendency against those with lower social status. Our study proposes a triadic framework of “perceived stimulus—meaning interpretation—aggressive behavior” to explain why displaced aggression occurs from a situational perspective. These findings align with classical studies and offer theoretical contributions to the current literature, while also acknowledging certain limitations. The following sections elaborate on the theoretical implications, practical relevance, and limitations of our research.

### 5.1. Interpretation: New Perspective of Displaced Aggression

The finding of H1 aligns with the classical frustration–aggression theory, which posits that suffering generates emotional tension that may be redirected toward weaker targets (Dollard et al., 2013; Marcus-Newhall et al., 2000). From the standpoint of reduced self-efficacy, perceived institutional inequality is associated with increased feelings of helplessness, which in turn encourages a downward aggression. According to control theory (Langer, 1975), when individuals feel a lack of agency in their environment, they may try to regain control through aggression. Such aggression is often directed toward accessible and less threatening targets rather than confronting powerful institutional actors. This displaced behavior is usually non-instrumental. It serves as a compensatory emotional regulation strategy that helps reduce distress and restore psychological balance. Although we acknowledge that frustration-induced negative affect can partially predict displaced aggression, a broader context calls for the involvement of meaning. Specifically, the assumption of homo narrans (Rabatel, 2008). We argue that self-motivation derived from interpretative meaning can effectively facilitate the emergence of aggressive behavior. Therefore, our study uses institutional inequality as the contextual framework, thereby broadening the theoretical scope of displaced aggression, and echoes the research of Bray et al. (2019) who highlight the symbolic factors in studies of aggressive behavior.

Specifically, the findings of H2 and H3 highlight the interpretation of intention in shaping behavioral responses to structural inequality. Traditional wisdom of general aggression model emphasizes the mediating function of high-arousal emotions, to predict the aggressive behaviors (Anderson & Bushman, 2002; Anderson & Bushman, 2018; C. T. Miller & Kaiser, 2001). Our study further suggests that specific perceived stimuli are more likely to shape a justification of institutional inequality, which in turn leads to displaced aggression. Our mediation analysis further indicates that when institutional inequality fosters the perception of lower aggression costs, both displaced and upward aggression tend to increase. In fact, some studies have pointed out that when the change in meaning pertains only to legitimacy, such change would be conservative in nature (Spiegler, 2020), as legitimacy is an abstract concept (Suddaby et al., 2017). This kind of shift tends to resonate only with audiences already attuned to the legitimacy narrative. However, when the change in meaning concerns the perceived cost of action, the shift becomes more radical (Shao & Liu, 2019). Because cost is a concrete and easily perceived concept, it often produces stronger spillover effects.

### 5.2. Authoritarian Reinforcement

H4 is a hypothesis that highly context-specific to China. Specifically, individuals with higher levels of pro-authoritarian attitudes are more likely to exhibit displaced aggression when their perceived institutional inequality is elevated. This aligns with the findings of Duckitt and Sibley (2007), who argue that authoritarian individuals display a heightened need to maintain social order and a strong tendency toward submission to authority. On the other hand, when this perceived order is threatened or when institutional equality is undermined, such individuals tend to experience defensive emotional arousal and develop stronger aggressive intentions toward those challengers (Bizumic & Duckitt, 2018). These dynamics are particularly pronounced in sociocultural contexts characterized by collectivism and high power distance. Importantly, displaced aggression among individuals with authoritarian personalities is not merely a result of submission to authority. It often reflects a proactive inclination to inflict harm, driven by a stronger hostile attribution bias (Milburn et al., 2014). From a political psychology perspective, one reason individuals adopt pro-authoritarian attitudes is a naïve belief in the inherent responsibility and legitimacy of the state or political institutions. This belief reinforces a positively biased image of the system, leading individuals to attribute social problems or disruptions not to institutional flaws, but to individuals deemed deviant or nonconforming (Glasius, 2018). As a result, hostility is displaced toward these individuals rather than directed at the system itself. Indeed, the scenarios assumed in existing literature typically involve negative issues that are broadly shared by the general public, such as economic crises (Sirotkina & Zavadskaya, 2020), or immigration crisis (Peresman et al., 2023), essentially representing low-intensity situation. In contrast, our study is grounded in the perceived institutional inequality, which directly targets an individual’s sense of personal injustice. This is not a universally experienced problem, but an extreme context that emphasizes personal relative deprivation.

To further interpret this pattern, it is essential to consider the broader cultural environment in which authoritarian orientations are cultivated and expressed. In collectivist societies, perceived institutional inequality is interpreted through normative frameworks that emphasize procedural legitimacy, relational harmony, and hierarchical stability rather than strict individual equality (Gouveia et al., 2002). Compared with individualist cultures, individuals in collectivist contexts display greater tolerance for unequal resource distribution (Huppert et al., 2019) and tend to prefer authority-mediated conflict resolution over direct, confrontational protest (Mattila & Patterson, 2004). These fairness judgments are embedded within cultural values that prioritize social order, in-group cohesion, and deference to authority, which in turn promote the internalization of beliefs such as the “just world” ideology, an attributional tendency to perceive inequality as legitimate or morally acceptable (Wu et al., 2011).

Within this cultural framework, pro-authoritarian attitude normalizes hierarchy-preserving emotion regulation and discourages direct confrontation with institutional power, which functions as a psychological manifestation of collectivist socialization. Consequently, individuals who endorse such orientations are more likely to redirect frustration toward socially permissible or weaker targets, thus amplifying the moderating effect of authoritarianism on displaced aggression. Meanwhile, collectivist norms reinforce the two cognitive mechanisms specified in our model. First, individuals learn to justify inequality by construing aggression against low-status others as a morally acceptable way to reaffirm harmony and relational order—corresponding to the inequality-justification pathway. Second, the same norms lower the perceived cost of aggression, as attacking weaker or out-group members is viewed as posing minimal relational risk, corresponding to the low-cost pathway. Together, these processes explain how perceived institutional inequality is cognitively reframed and behaviorally displaced within collectivist contexts (Dang et al., 2025).

### 5.3. Gendered Dynamics

Our study further reveals that gender significantly moderates the relationship between perceived institutional inequality and displaced aggression, particularly in the domains of displaced aggression and emotional suppression. Compared to male participants, females are more likely to engage in displaced aggression when perceiving institutional inequality. Although traditional theories of social aggression suggest that hormone levels, social expectations, and gender norms contribute to higher levels of aggression among men (Björkqvist, 2018), our findings appear to contradict this pattern. However, by further disaggregating the forms of aggressive behavior, we uncovered evidence that departs from conventional assumptions. We argue that these results remain consistent with the theoretical foundations of existing research; in fact, females often face greater social expectations than males, which may make them more likely to engage in displaced aggression. For example, research has shown that female leaders in traditional organizations do not significantly favor female subordinates (even discriminate them), in part to avoid the appearance of political correctness (Derks et al., 2016; Flabbi et al., 2019). This echoes the foundational claims of gender socialization theory, which posits that males are socialized with overt agency and assertive behavior, while females are more often socialized toward relational harmony and emotional restraint (Crick & Grotpeter, 1995; Eagly & Wood, 2012). Furthermore, when females engage in upward resistance or aggression in public settings, they tend to face greater social pressure. As a result, they may shift toward forms of aggression that involves lower social costs, such as displaying “resentment” to people within one’s social circle. As emotion regulation theory indicates that females are more likely to suppress or transform emotions, whereas males tend to release emotional arousal through external behaviors (Gross & John, 2003). In addition, males have greater access to public resources to cope with institutional inequality, which offsets their motivation for upward protest, especially within a high-pressure social environment (Acker, 2006). All these explanations may account for why females exhibit stronger tendencies toward displaced aggression when perceiving institutional inequality. On this basis, our finding suggest that females are, to some extent, “compelled” to adopt displaced aggression as a mechanism for relieving perceptual stress.

### 5.4. Contributions and Implications

Our study offers three theoretical contributions. First, in explaining displaced aggression, we argue that inequality, especially institutional deprivation, offers broader explanatory validity. Traditional theories of displaced aggression often attribute the phenomenon to proximal causes such as frustration encounters, stimulus generalization, hostile responses, or behavioral learning (Bandura, 2024; Denson et al., 2006; Lagios et al., 2025; Marcus-Newhall et al., 2000; N. Miller et al., 2003). These explanations suggest a structural mismatch, in which the aggressor typically targets a low-risk scapegoat while the true source of stress remains out of reach. This dynamic reflects an underlying power asymmetry between social groups. Therefore, we use the perceived institutional inequality as the explanatory variable and introduce a three-level relational framework, comprising “the institution (higher-status actor), the individual, and subordinates (lower-status targets),” to build a more robust and contextually grounded theory.

Second, from a methodological perspective, our research offers a more contextualized and interdisciplinary explanation. By employing a scenario-based survey, we embed the explanatory framework within specific situational contexts, rather than relying on abstract opinion-based questions as in traditional surveys. This approach enhances external validity while allowing for more accurate identification of contextual effects. Moreover, our design places greater emphasis on mechanism-based explanations. We argue that a single informational stimulus is insufficient condition for triggering aggressive behavior. Perceived institutional inequality must be interpreted in a way that activates aggression-related intentions. In this respect, our approach adopts a narrative paradigm to reinterpret a classic topic in social psychology. Furthermore, by incorporating the moderating roles of pro-authoritarian attitude and gender, we propose a comprehensive explanatory framework that integrates insights from political science and sociology. This allows the theory of displaced aggression to maintain its explanatory validity in more complex social environments.

Third, this study extends the theoretical boundaries of displaced aggression by demonstrating its robustness under a more targeted, psychologically acute form of stress. Whereas previous research has primarily examined displaced aggression in response to diffuse or impersonal macro-level shocks, such as economic crises (Sirotkina & Zavadskaya, 2020). Our key predictor—perceived institutional inequality—captures a more targeted, affectively intense, and inescapable form of structural stress. Unlike broad structural stressors that often lead to cynicism or political disengagement (Shao & Liu, 2019), this micro-level perception of injustice triggers more direct behavioral expressions of aggression, even in high self-control context like China. These findings suggest that displaced aggression functions as a universal phenomenon, capable of manifesting not only under liberal-democratic frustrations but also within repressive political environments. This contributes to a more nuanced understanding of how perceived institutional inequality transforms into misdirected aggression, regardless of the surrounding regime type.

Our study also offers two practical implications for policymakers. First, we emphasize that displaced aggression is not merely a consequence of social frustration, but also of broader institutional inequality and the justification it promotes; that is, inequality among people is acceptable. Therefore, policymakers must pay greater attention to the narrative behind social problems. Second, our findings show that certain social groups, such as those with pro-authoritarian orientations (or broadly conservative) and females, are more susceptible to engaging in displaced aggression. This suggests that effective governance of social aggression should be approached from broader perspectives such as traditionalism and patriarchy, in order to address its deeper structural roots.

### 5.5. Limitations and Outlook

Nevertheless, our study has certain limitations, which also point to potential directions for future research. First, our study relies on survey data. While this is a mainstream method in behavioral science research, we acknowledge that the questionnaire format may lead to potential measurement bias. Future research could incorporate observational data, such as users’ social media activity, to analyze patterns of social aggression more comprehensively. Second, our study focuses exclusively on China and uses a sample of university students. While China presents a unique sociocultural environment that is well-suited for studying displaced aggression, the reliance on student participants within a single national context may limit the generalizability of the findings. Future research could address this limitation through cross-national surveys and comparative studies that include more diverse and non-student populations. Third, we did not employ experimental research because it may not satisfy our scenario-based demand. Due to ethical constraints, it is not feasible to create a scenario in which participants engage with a real target of aggression in a lab. This may lead to measurement errors if we employed experimental research, where expressions of dissatisfaction are misinterpreted as aggression. Future research could employ tools such as eye-tracking or electroencephalography (EEG) to capture physiological indicators of aggression, thereby improving the feasibility and precision of experimental approaches.

## 6. Conclusions

Our study seeks to explain the widespread occurrence of displaced aggression. Unlike traditional theories, which often focus on single social factors or static informational stimuli to account for behavioral outcomes, we propose a multidimensional explanatory framework for social aggression. Central to our framework is the concept of structural misalignment of social status, referring to the perceived asymmetry in which some individuals are viewed as more “attackable” than others. Using a scenario-based survey, we measured this structural misalignment through perceived institutional inequality, incorporating multiple contextual dimensions such as superiors, the state, and society. Our results demonstrate that this structural misalignment significantly contributes to the emergence of displaced aggression. Moreover, systemic inequality implies a socially sanctioned belief in inequality, which lowers the perceived cost of aggressive behavior and thus facilitates displaced aggression. Furthermore, we find that individuals with pro-authoritarian attitude and female participants are prone to engage in displaced aggression. This highlights the close ties between aggressive behavior and broader political ideologies, as well as the social resilience shaped by gender dynamics. Together, our study offers a narrative-based explanatory perspective on social aggression and proposes an interdisciplinary interpretive framework.

## Figures and Tables

**Table 1:** (**a**). Descriptive Statistics for Continuous Variables. (**b**). Descriptive Statistics for Categorical Variables.

**(a)**		
**Key Variables**	**Mean**	**SD**
Displaced Aggression	4.21	0.99
Upward Aggression	2.43	0.80
Perceived institutional Inequality	3.32	0.57
Awareness of Lower Aggression Cost	3.05	0.96
Inequality Justification	2.50	0.87
Pro-authoritarian Attitude	2.74	0.97
Age	22.47	1.65
Yearly Family Income (CNY)	132,789.12	2783.85
Observations	1109	
**(b)**		
**Key Variables**	**N**	**Percentage (%)**
Gender (Female)	409	37%
CCP Membership	233	21%
Household registration (Urban)	811	73%
Observations	1109	

Note: (a) This table reports descriptive statistics for all key variables included in the analysis. Values are sample means and standard deviations (SD). All other variables are measured on 5-point Likert-type scales. (b) This table reports descriptive statistics for all key categorical variables included in the analysis. Frequencies (N) and percentages are presented. All percentages are rounded to one decimal place.

**Table 2:** Effects of Perceived Institutional Inequality on Aggressive Responses.

	Column 1	Column 2
Dependent Variable	Displaced Aggression	Upward Aggression
Perceived Societal Unfairness	0.388 ***	−0.091
	(0.048)	(0.049)
Constant	3.097 ***	2.667 ***
	(0.192)	(0.185)
Control Variable	YES	YES
Adjusted R^2^	0.054	0.005
Observations	1109	

Note: Standard errors are reported in parentheses. Dependent variables include displaced aggression, upward aggression, and emotional suppression. *** *p* < 0.001.

**Table 3:** Mediation Effect via Awareness of Lower Aggression Cost.

	Column 1	Column 2
Dependent Variable	Displaced Aggression	Upward Aggression
IV on Mediator	Significant	Significant
Mediator on DV	0.156 ***	0.056 *
Proportion mediated	15.3%	23.7%
Observations	1109	

Note: Mediator on DV represents the estimated effect of awareness of lower aggression cost on each dependent variable. Proportion mediated refers to the share of the total effect accounted for by the indirect path. IV on mediator means the significance of perceived institutional inequality on awareness of lower aggression. All models have control variables as H1. * *p* < 0.05, *** *p* < 0.001.

**Table 4:** Mediation Effect via Inequality Justification.

	Column 1	Column 2
Dependent Variable	Displaced Aggression	Upward Aggression
IV on Mediator	Significant	Significant
Mediator on DV	0.155 ***	0.046
Proportion mediated	12.4%	N/A
Observations	1109	

Note: Mediator on DV represents the estimated effect of inequality justification on each dependent variable. Proportion mediated refers to the share of the total effect accounted for by the indirect path. IV on mediator means the significance of perceived institutional inequality on awareness of lower aggression. All models have control variables as H1. N/A = Not applicable. *** *p* < 0.001.

**Table 5:** Moderating Effects of Pro-Authoritarian Attitude.

	Column 1	Column 2
Dependent Variable	Displaced Aggression	Upward Aggression
Interaction Term (Pro-Authoritarianism)	0.224 ***	−0.065
	(0.035)	(0.049)
Constant	5.385 ***	2.273 ***
	(0.298)	(0.491)
Control Variables	YES	YES
Adjusted R^2^	0.111	0.011
Observations	1109	

Note: Standard errors are reported in parentheses. All models include the main effects of perceived institutional inequality and pro-authoritarian attitude, along with their interaction term. Dependent variables include displaced aggression and upward aggression. All models have control variables and robust standard errors setting same as the H1. *** *p* < 0.001.

**Table 6:** Moderating Effects of Gender.

	Column 1	Column2
Dependent Variable	Displaced Aggression	Upward Aggression
Interaction Term (Female = 1)	0.248 *	−0.128
	(0.102)	(0.104)
Constant	3.162 ***	2.597 ***
	(0.194)	(0.199)
Control Variables	YES	YES
Adjusted R^2^	0.059	0.008
Observations	1109	

Note: Standard errors are reported in parentheses. All models include the main effects of perceived institutional inequality and gender binary variable (female = 1), along with their interaction term. Dependent variables include displaced aggression and upward aggression. All models have control variables and robust standard errors setting same as the H1. * *p* < 0.05, *** *p* < 0.001.

**Figure A2:**
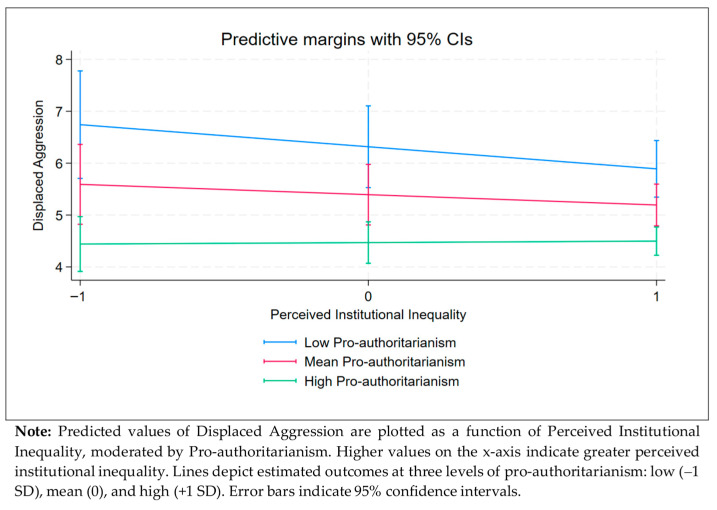
Interaction Between Perceived institutional inequality and Pro-authoritarian Attitude on Displaced Aggression.

**Figure A3:**
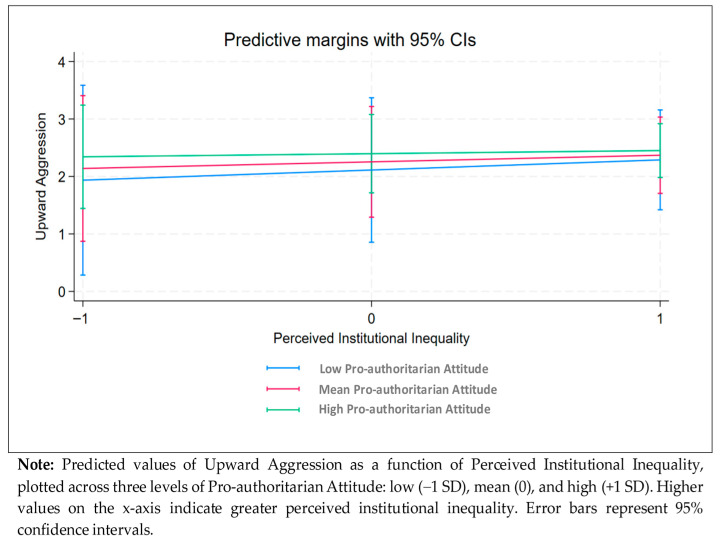
Interaction Between Perceived institutional inequality and Pro-authoritarian Attitude on Upward Aggression.

**Figure A4:**
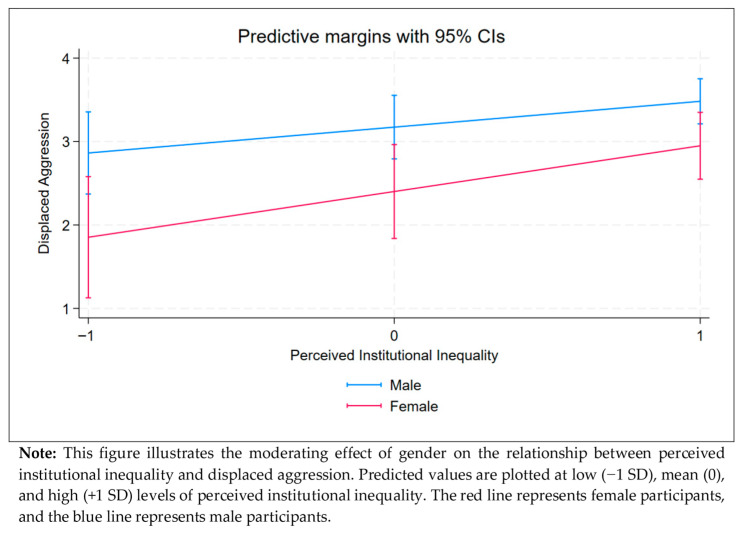
Interaction Between Perceived institutional inequality and Gender on Displaced Aggression.

**Figure A5:**
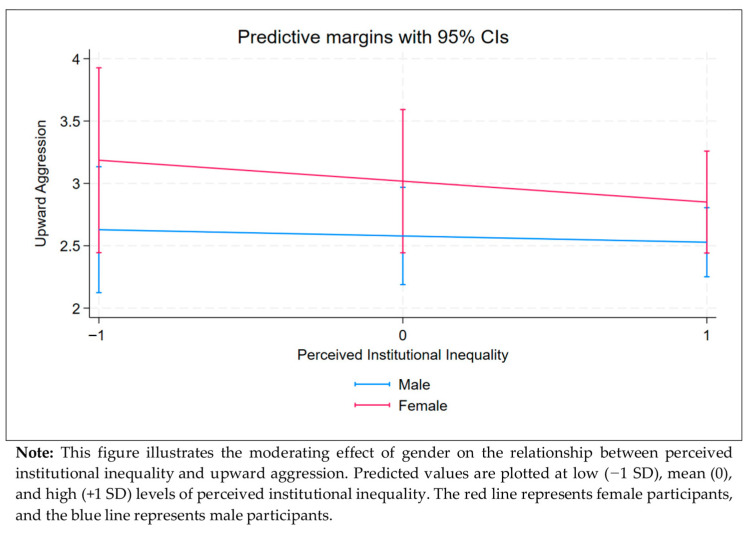
Interaction Between Perceived institutional inequality and Gender on Upward Aggression.

## Data Availability

If data and codes are required then they can be obtained by contacting the corresponding author or the first author.

## Appendix A

### Pearson Correlation Matrix for Key Variables

**Table A1:** Pearson Correlations Among Key Variables.

Variables	(1)	(2)	(3)	(4)	(5)	(6)	(7)	(8)	(9)	(10)	(11)
Perceived Societal Unfairness	1.000										
Displaced Aggression	0.226 ***	1.000									
Upward Aggression	−0.067 *	−0.051	1.000								
Awareness of Lower Aggression Cost	−0.205 ***	0.085 ***	0.060 *	1.000							
Inequality Justification	−0.223 ***	0.092 **	0.077 *	0.960 ***	1.000						
pro-authoritarian attitude	−0.256 ***	0.129 ***	0.0751 *	0.289 ***	0.316 ***	1.000					
Gender	0.001	0.010	0.290	−0.053	−0.049	−0.012	1.000				
CCP Membership	0.028	0.041	−0.073 *	0.013	0.017	0.004	0.013	1.000			
Age	−0.115 ***	−0.020	0.061 *	−0.054	−0.034	0.040	−0.017	0.021	1.000		
Household Registration	−0.075 *	−0.083 **	0.044	0.013	0.014	0.045	−0.071 *	0.001	0.142 ***	1.000	
Annual Household Income (in RMB)	0.053	0.067 *	−0.023	0.186	0.023	−0.038	0.055	0.046	−0.071 *	−0.074 *	1.000

Note: This table reports Pearson correlation coefficients among the main variables. N = 1109. All variables are continuous unless otherwise noted. * *p* < 0.05, ** *p* < 0.01, *** *p* < 0.001.

## Appendix C

### Appendix C.1. Robustness Check 1: Sensitivity Analysis

**Table A2:** Sensitivity Analysis with Benchmarking on CCP Membership.

Variable	DV	Coef.	S.E.	RV_q	Bound (CCP Membership)	Bound Coef.	Lower CI	Upper CI
CCP Membership	Displaced Aggression	0.3914	0.0510	0.2057	1.00×	0.3897	0.2896	0.4898
					2.00×	0.3880	0.2880	0.4881
					3.00×	0.3863	0.2863	0.4863
	Upward Aggression	−0.0906	0.0420	0.0627	1.00×	−0.0878	−0.1701	−0.0054
					2.00×	−0.0850	−0.1671	−0.0028
					3.00×	−0.0821	−0.1641	−0.0002

Note: This table reports sensitivity analysis results assessing the robustness of estimated effects to potential omitted variable bias. RV_q indicates the minimum strength an unobserved confounders would need to nullify the treatment effect. Results show the effects remain robust even if unobserved confounders are 2–3 times stronger than the benchmark.

### Appendix C.2. Robustness Check 2: Including Grade and Major as CV

**Table A3:** Regression with Additional Control Variables.

Dependent Variable	Displaced Aggression	Upward Aggression
Perceived Institutional Inequality	0.471 ***	−0.056
	(0.520)	(0.044)
R^2^	0.094	0.011
Control Variable	Yes	Yes
Additional Control Variable	Yes	Yes
Constant	3.447 ***	2.600 ***
	(0.253)	(0.214)
Observations	1109	

Note: Total observation number is 1109. OLS regressions include additional control variables academic grade and major, to assess the robustness of the main effects. Robust standard errors are reported in parentheses. Results remain statistically significant and consistent, indicating that the estimated effects are stable after accounting for these covariates. *** *p* < 0.01.

### Appendix C.3. Robustness Check 3: Change IV with Pro-Authoritarian Attitude

**Table A4:** Change Perceived institutional inequality with Pro-authoritarian Attitude.

Dependent Variable	Displaced Aggression	Upward Aggression
Pro-authoritarianism	−0.132 ***	−0.062 **
	(0.030)	(0.025)
Observations	1109	1109
R^2^	0.017	0.009
Control Variable	Yes	Yes
Constant	4.623 ***	2.652 ***
	(0.104)	(0.084)

Note: Total observation number is 1109. All regressions are estimated using OLS with robust standard errors. Robust standard errors are reported in parentheses. Results remain statistically significant and consistent. ** *p* < 0.05, *** *p* < 0.01.

### Appendix C.4. Use Ordered Logistic Regression (Ologit)

**Table A5:** Robustness Check Using Ordered Logistic Regression.

Dependent Variable	Displaced Aggression	Upward Aggression
Perceived Institutional Inequality	1.051 ***	−0.160
	(0.105)	(0.106)
Observations	1109	1109
z	9.94	−1.51
R^2^	0.028	0.003
Control Variable	Yes	Yes
Log likelihood	−2047.626	−1986.377

Note: This table reports the results of ordered logistic regression (ologit) models used for robustness checks. Coefficients represent the log-odds of higher levels of the outcome variable. Standard errors are in parentheses. *** *p* < 0.01.
